# Sudden death as presenting symptom caused by cardiac primary multicentric left ventricle rhabdomyoma, in an 11-month-old baby. An immunohistochemical study

**DOI:** 10.1186/1746-1596-7-169

**Published:** 2012-12-03

**Authors:** Margherita Neri, Sabina Di Donato, Rocco Maglietta, Cristoforo Pomara, Irene Riezzo, Emanuela Turillazzi, Vittorio Fineschi

**Affiliations:** 1 Department of Forensic Pathology, University of Foggia, Ospedale Colonnello D’Avanzo, Via degli Aviatori 1, Foggia, 71100, Italy; 2 ASL BAT, Ospedale di Barletta, Barletta, 76121, Italy; 3 ASL, Ospedale di Matera, Matera, 75100, Italy

**Keywords:** Cardiac rhabdomyoma, Sudden death, Cardiac tumours, Paediatric tumours

## Abstract

This case report describes a sudden cardiac death in an apparent healthy 11-month-old infant caused by a multifocal cardiac rhabdomyoma. Parents reported that a few days before the child had fallen to the ground getting a little superficial injury to the scalp. The authors hypothesize that it may have been a transient loss of consciousness episode caused by the cardiac tumour. After the gross examination, histological investigation supported by immunohistochemical analysis using antibody anti- Myoglobin, Actin, Vimentin, Desmin, CD34, S-100, Ki-67 was carried out for the diagnosis. Death was attributed to a multifocal cardiac rhabdomyoma, a benign tumour of striated muscle, which has been completely asymptomatic. In particular, one mass filled the entire posterior wall of the left ventricle. The insidious development of benign cardiac tumours also in infants and children is outlined, focusing on the responsible mechanisms of sudden death in such cases and providing a reference for additional study on these subjects.

**Virtual slides:**

The virtual slide(s) for this article can be found here:

http://www.diagnosticpathology.diagnomx.eu/vs/7163626988365078

## Background

Cardiac tumours can be divided into primary and secondary; the first ones occur infrequently both in adult and paediatric age groups. In the general population, their incidence ranges between 0.001% and 0.030% in unselected autopsy series; approximately 75% of primary cardiac tumours are benign, and 25% are malignant [1]. In adults the most common ones are myxomas.

In the paediatric population, cardiac tumours are reported with a frequency of 0.02% to 0.04% [2,3]. Usually they are benign, with minimal growth potential, but a minor portion can become quite large, compressing the cardiac chambers or the vital structures such as conduction tissue or coronary blood vessels, as well as obstructing the cardiac valves and the outflow tracts.

Most paediatric cardiac tumours are considered benign; however, the development of hemodynamic impairment or malignant arrhythmias may complicate the management of these tumours in some patients. In particular, malignant arrhythmias increase the risk of sudden cardiac death [4]. In the absence of these complications, observation is the standard care in most cases because many tumours spontaneously regress with time.

We report the case of a sudden cardiac death of a 11-month-old female infant with a primary multicentric left ventricle tumour. Histological and immunohistochemical studies allowed to make the diagnosis of rhabdomyoma and to define the mechanism inducing sudden death. This report outlines the importance of studies performed during the third trimester of pregnancy for early diagnosis of cardiac tumours which, although histologically benign, may be life-threatening for the arrhythmogenic potential they display.

## Case presentation

A mother was bathing her 11-month-old baby, but suddenly the infant showed a worsening dyspnoea. Parents accompanied the baby to the emergency room immediately, but despite the resuscitative manoeuvres, the doctor could only pronounce the death. The familiar history was negative for sudden death. Also the obstetric, remote and recent pathological anamneses were negative, except for a referred unexplained fall dating back to two days before.

A complete post-mortem examination was performed within 48 hours after death. The body was that of a regularly developed 11-month old infant. External examination was insignificant, except for the presence of a little and superficial iatrogenic wound on the sternal region.

**Figure 1:**
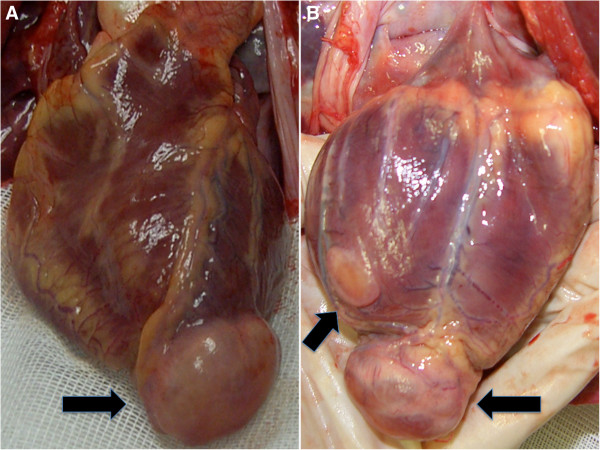
**Microscopic findings.** The peduncolated mass at the cardiac apex measured 4,5 × 3 × 2cm (**A**). A second superficial subepicardial neoformation on the posterior wall of the left ventricle, measured 2 **×** 1,5 **×** 1cm (**B**).

The internal examination revealed a peduncolated 4,5 × 3 × 2 cm mass at the cardiac apex, a second superficial subepicardial 2 × 1,5 × 1 cm neoformation on the posterior wall of the left ventricle and a third transmural 1 × 1,2 × 1,4 cm nodule on the posterior wall of the left ventricle (Figure 1). Polyvisceral congestion, cerebral and pulmonary oedema, with a massive increase in lung weight were also evident.

The histological examination of cardiac specimens, stained with haematoxylin–eosin, Masson and PTAH showed a demarcation and separation of the three masses from the surrounding regular parenchyma. The striated muscle cells appeared diffusely vacuolated, enlarged, with round to oval slightly irregular nuclei and variable cytoplasmatic clearing. There were occasional spider cells. Small residues of muscular tissue entrapped in the masses were also visible.

In addition an immunohistochemical investigation of cardiac tissue samples was performed utilizing antibodies anti-Myoglobin, Actin, Vimentin, Desmin, CD 34, Ki67 and S100 (DAKO, Copenhagen, Denmark). For this study, we used 4 μm-thick paraffin sections mounted on slides covered with 3, aminopropyltriethoxysilane (Fluka, Buchs, Switzerland). Pre-treatment was necessary to facilitate antigen retrieval and to increase membrane permeability to antibodies anti-CD 34 boiling in 0.25 M EDTA buffer, to antibodies anti-Ki67 boiling in 0.1 M Citric Acid buffer, to antibody anti-S100 for 15 min in Proteolytic Enzyme (Dako, Copenhagen, Denmark), at 20°C. For antibody anti- Myoglobin, Actin, Vimentin, Desmin it wasn’t necessary a pre-treatment for antigen retrieval. The primary antibody was applied in 1:300 ratio for S100, in 1:50 ratio for CD 34, Desmin and Vimentin, in 1:2000 ratio for Actin, in 1:6000 ratio for Myoglobin, in 1:100 ratio for Ki67 and incubated for 120 min at 20°C. The detection system utilized was the LSAB+ kit (Dako, Copenhagen, Denmark), a refined avidin-biotin technique in which a biotinylated secondary antibody reacts with several peroxidase-conjugated streptavidin molecules. The sections were counterstained with Mayer’s haematoxylin, dehydrated, cover slipped and observed in a Leica DM4000B optical microscope (Leica, Cambridge, UK).

**Figure 2:**
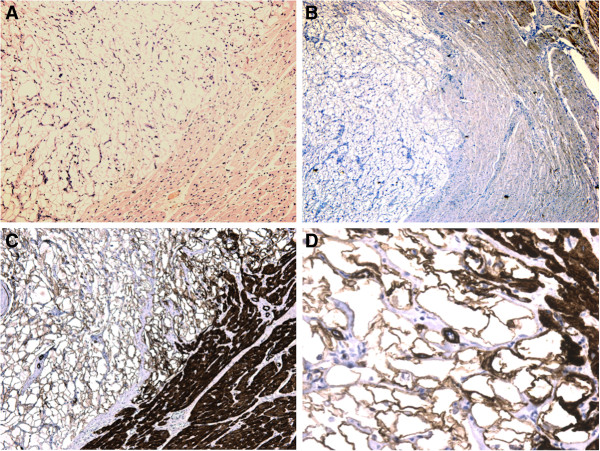
**Microscopic findings.** (**A**) Demarcation and separation of the tumoral mass from the surrounding regular parenchyma. Small residues of muscular tissue entrapped in the masses were also visible. Intense positive reaction for antibodies anti-Desmin (**B**), anti-Actin (**C**-**D**).

**Figure 3:**
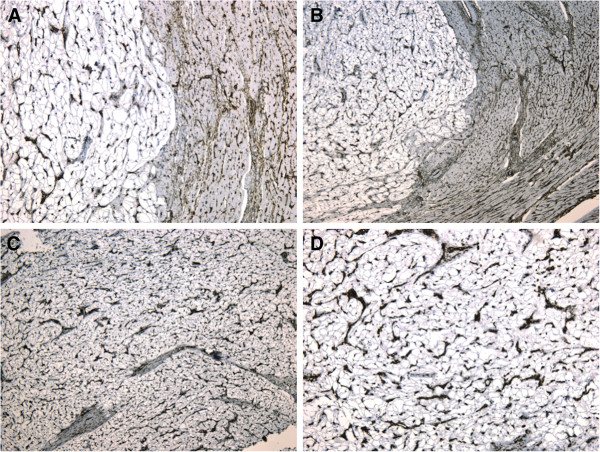
**Microscopic findings.** (**A**-**B**) Intense positive reaction of the tumoral fibers for antibodies anti-CD34 and anti-Vimentin (**C**-**D**).

The immunohistochemical studies showed an intense positive reaction for antibodies anti-Myoglobin, Actin, Vimentin, Desmin, CD34; while reaction for antibodies Anti-Ki67 and Anti-S100 was negative (Figures 2–3). These results of the immunohistochemical analysis were consistent with the diagnosis of rhabdomyoma.

Toxicological screening resulted negative. There were no signs of tuberous sclerosis.

It was concluded that the sudden cardiac death of the infant was attributable to the multifocal primary cardiac tumour, the rhabdomyoma, consisting in three cardiac lesions. In particular one tumoral mass occupied almost the whole posterior wall of the left ventricle, rising from the apex to the valvular level, so compromising the regular contraction of the left ventricle. The neoplasms probably have caused two days before a near syncopal episode that the parents erroneously referred as a fall.

## Conclusions

Cardiac tumours are infrequent clinical entities both in adult and paediatric age groups; they can be divided into primary and secondary; approximately 75% of primary cardiac tumours are benign, and 25% are malignant [1]. The occurrence of metastatic cardiac tumours has been reported 100-fold more commonly than primary lesions. Approximately half of benign cardiac tumours in adults are myxomas, and the rest are lipomas, papillary fibroelastomas, and rhabdomyomas. Among malignant primary cardiac tumours, the most reported are those histopathologically considered as undifferentiated, followed by angiosarcomas and leiomyosarcomas.

Cardiac tumours in children are rare. It is difficult to estimate the real prevalence because the available data are restricted to autopsy and case records from leading paediatric centres. It is believed that they constitute less than 0.1% of all neoplasms and occur with an incidence of 0.0017 to 0.28 in autopsy records [5,6].

Among 27.640 patients examined echocardiographically in 5-year periods, the incidence of cardiac tumours increased: 0.06% (1980–1984), 0.22% (1985–1989) and 0.32% (1990–1995) of all the subjects [7].

Benign lesions usually predominate, making up more than 90% of all paediatric tumours: rhabdomyomas are the most frequent cardiac tumours in children (45-50%), followed by fibromas (25-30%), mixomas, lipomas, teratomas, hemangiomas, etc. [8-10]. The most common malignant primary tumours are angiosarcoma, fibrosarcoma, lymphosarcoma, and giant cell sarcoma [2]. Secondary cardiac neoplasms in the paediatric population include non-Hodgkin lymphoma, leukemic infiltration, and neuroblastoma.

Rhabdomyoma is the most common cardiac tumour in paediatric patients. It usually presents during the first few days after birth, but it can be diagnosed in the third trimester of pregnancy.

Foetal cardiac rhabdomyoma accounts for less than 10% of foetal demise cases [11]. Cardiac rhabdomyomas are benign from the cardiovascular standpoint in most affected foetuses. They are associated strongly with tuberous sclerosis, a hereditary disorder characterized by hamartomas in various organs, epilepsy, mental deficiency, and sebaceous adenomas [12]. Rhabdomyoma (especially multiple) affects about 50% of children suffering from tuberous sclerosis, but more than 50% of patients with rhabdomyoma have or will develop tuberous sclerosis. The exceptional patient is one with a solitary, single rhabdomyoma who does not have or develop tuberous sclerosis.

Over 90% of rhabdomyomas are multiple and occur with approximately equal frequency in both ventricles. The atrium is involved in fewer than 30% of patients.

Macroscopically, these tumours are firm, grey, and nodular and tend to project into the ventricular cavity. Microscopy shows myocytes of twice normal size filled with glycogen and containing hyperchromatic nuclei and eosinophilic-staining cytoplasmic granules. Scattered bundles of myofibrils can be seen within cells by electron microscopy.

These tumours grow during the second half of pregnancy; they have the tendency to diminish or even completely disappear spontaneously after birth. Many will disappear entirely; alternatively, the tumour size remains constant as the heart grows, which has much the same effect [13,14].

In the majority of cases cardiac rhabdomyomas are clinically completely silent and have a benign course. They may be incidentally discovered during an echocardiogram, but they also may cause cardiac dysfunctions requiring medical and/or surgical intervention. During foetal life and the early neonatal period, life-threatening conditions, mostly due to arrhythmias, cardiac failure or obstruction, do occur on rare occasions [15]. How threatening rhabdomyomas are, in terms of clinical presentation and presence of hemodynamic complications, depends on their size, location and number of lesions. Although the behaviour of cardiac rhabdomyoma is benign, its positioning within critical areas in the heart can lead to lethal arrhythmias and chamber obstruction. The most common presentation is heart failure caused by tumour obstruction of cardiac chambers or valvular orifice flow. Clinical findings may mimic valvular or subvalvular stenosis. Arrhythmias, particularly ventricular tachycardia and sudden death, may be a presenting symptom [16,17]. Atrial tumours may produce atrial arrhythmias. The mechanism of their causing sudden death is likely rhythm disturbances, particularly the Wolff-Parkinson-White ventricular pre-excitation syndrome [17]. The conduction abnormalities and the arrhythmia are thought to be due to the tumoral mass, which may lead to degeneration of the cardiac conduction system and compose a substrate for re-entrant arrhythmia, e.g. the development of atrioventricular block and ventricular arrhythmia. Large tumoral mass in the myocardial region, accompanied with arrhythmia, revealed that the neoplastic mass could affected the common bundle of His, in addition to the ordinary myocardium, being causative of circulatory failure [18,19].

Although most cardiac rhabdomyomas have a relatively benign perinatal course, the long-term prognosis is determined by the neurological manifestations associated with tuberous sclerosis.

Rhabdomyoma may be detected prenatally by ultrasound in the third trimester of pregnancy. However, a large number is diagnosed in infancy. The diagnosis is suggested by clinical features of tuberous sclerosis and is made by echocardiography. Heart echocardiography, CT and MRI are the major non-invasive diagnostic procedures, while angiography is used in selected cases, due to its invasive nature. Even though ECG is not specific for the diagnosis, it is important to establish the presence of rhythm disorders, while a chest X-ray may show an enlarged heart silhouette or abnormal cardiac contours.

Therapeutic strategies should be individualised: the total resection is not the only therapeutic aim, in fact, the restoration of the regular heart function may be of primary relevance. Antiarrhythmic agents successfully control the clinical and electrophysiological conditions, while surgery is indicated in children with significant clinical symptoms. Benign cardiac tumours in childhood have an excellent prognosis when completely excised and have a good short-term prognosis even when excision is incomplete. Asymptomatic patients require anyway a close follow-up [20].

This report outlines the importance of an early diagnosis of cardiac tumours: when the prenatal and/or neonatal diagnosis of rhabdomyoma is made, an appropriate planning and an accurate follow-up may be performed to formulate individualised therapeutic strategies, improving in this way the clinical outcome and, eventually, anticipating the development of tuberous sclerosis.

## Consent

Written informed consent was obtained from the patient’s parents for publication of this case report and any accompanying images.

## Competing interests

The authors declare that they have no competing interests.

## Authors' contributions

VF contributed to this article and conceived the study. SDD and ET wrote the manuscript. RM and CP performed the macroscopic sections. MN and IR made the pathological explorations and performed the laboratoristic and microscopic analysis. All authors read and approved the final manuscript.
